# Preparation and Characterization of Polypropylene/Sepiolite Nanocomposites for Potential Application in Automotive Lightweight Materials

**DOI:** 10.3390/polym15040802

**Published:** 2023-02-05

**Authors:** Guofeng Wu, Liang Lei, Yijian Wu, Fei Yu, Jianjun Li, Hui He

**Affiliations:** 1 School of Materials Science and Engineering, South China University of Technology, Guangzhou 510640, China; 2 National-Certified Enterprise Technology Center, Kingfa Science and Technology Co., Ltd., Guangzhou 510663, China

**Keywords:** polypropylene, sepiolite, nanocomposites, automotive, lightweight

## Abstract

Polypropylene (PP)/sepiolite nanocomposites were prepared using the melt blending technique. The effects of nano-sepiolite content on the mechanical property, thermal property, crystallinity, morphology and rheological property of PP/sepiolite nanocomposites were investigated. The organic modified sepiolites (OSep) were dispersed evenly in PP matrix after surface treatment. The addition of OSep improved the storage modulus and thermal stability, showing a strong interaction between OSep and PP matrix. With the increase of OSep content, the fluidity of PP/OSep composites first increased due to the lubrication of surface modifiers and then decreased due to the interaction between OSep and PP. The size of the toughening agent elastomer first increased and then decreased, and the impact notched strength of PP/Osep composites first decreased and then increased. The loading of OSep also reduced the crystallinity and shrinkage rate of PP. PP/OSep nanocomposites have potential applications in high-performance automotive lightweight materials.

## 1. Introduction

Polypropylene (PP) has been widely used in the packaging, automotive, medical and textile industries in the last few decades because of its outstanding processing performance, mechanical properties and low cost. For the automotive industry, PP materials are very common in the interior and exterior parts. Automotive materials generally require a good balance between rigidity and toughness. In order to improve the toughness of PP materials, thermoplastic olefin elastomer (POE) is usually used as a toughening agent. However, the POE loading will reduce the rigidity of PP materials. Inorganic fillers, such as talc, glass fiber and calcium carbonate, are widely used to improve the rigidity of PP materials. At present, the loading of fillers is usually up to 20 wt%, leading to a high density. However, lightweight is one of the most important directions of automotive industry development. The density reduction, thin-wall design and introduction of foaming structures are the main three methods to realize the lightweight of automotive materials. Reducing filler content is one commonly used way to reduce the density and weight of automotive materials. However, the reduction of filler loading will increase the mold shrinkage of materials, which leads to the dimensional mismatching between the original mold and the final parts. Therefore, the dimensional accuracy of automotive parts using low density materials is a challenge.

In recent years, researchers have focused their attention on the nanocomposites such as nano-clay [1,2], carbon nanotube [3,4,5,6], graphene [7] and so on. The nano-clay is one of the most promising nano-fillers for industrial application due to its low price and obvious enhancement effect. The most commonly used nano-clays are montmorillonite (MMT) [8,9,10,11,12], sepiolite [13,14,15], attapulgite [16,17,18], halloysite [19,20,21], etc. The addition of nano-fillers in the polymer matrix can bring some advantages such as improved mechanical properties, thermal stability, fire resistance, gas barrier, etc.

For PP/MMT nanocomposites, MMT achieves a very good enhancement effect only when MMT is fully exfoliated. However, it is difficult to exfoliate MMT nanosheets because MMT is a two-dimension nano-filler with high specific surface area. So, MMT is still not widely used in modified plastics. Sepiolite is a needle-like fibrous hydrated magnesium silicate clay mineral with layer chain structure. Sepiolite has a very high surface area, as high as 200–300 m^2^/g, a length of 0.2–4 μm, a width of 10–30 nm and a thickness of 5–10 nm [13]. Due to this unique morphology, polymer chains can not only interact with the external surface of sepiolite, but also penetrate into the internal structure, which facilitates a uniform dispersion of sepiolite in the polymer matrix. In previous studies on PP/sepiolite nanocomposites, the compatibilizer (maleic anhydride grafted-polypropylene (PP-g-MA) is used for improving the dispersion of sepiolite in PP matrix. The mechanical properties of PP/sepiolite nanocomposites are improved after adding PP-g-MA compared to those without using compatibilizers. In addition, the sepiolite can play a synergistic flame retardant effect in flame retardant applications. So, the flame retardant effect could be improved in flame retardant PP/sepiolite composites. However, previous studies have been conducted in neat PP resin without the second component. Automotive PP materials require a certain toughness, and usually contain elastomer toughening agents. The nano-sepiolite can also be organically modified to improve the compatibility and dispersibility of nano-sepiolite and PP, instead of adding the compatibilizer PP-g-MA.

**Figure 1:**
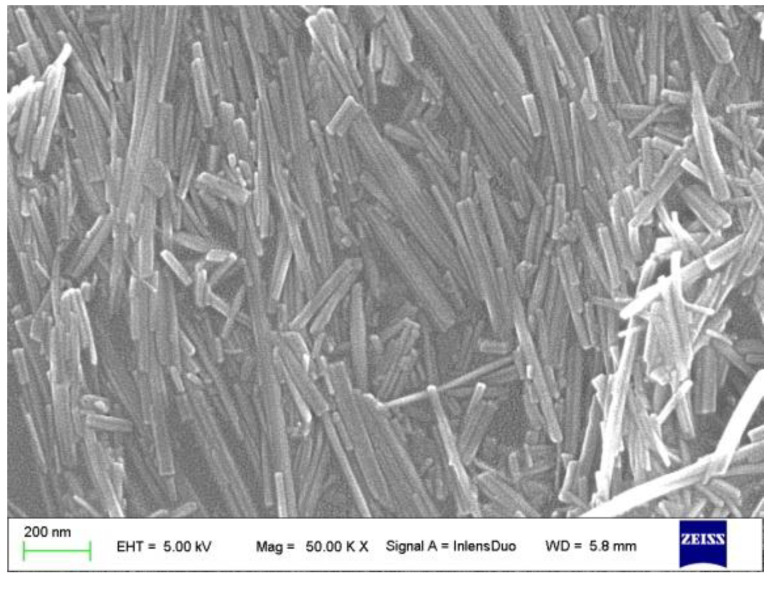
Morphology of nano-sepiolite.

Therefore, this research aims to improve the performance of PP containing elastomers through melt blending with organically modified nano-sepiolite (OSep) as a reinforcing filler. The morphology of nano-sepiolite is shown in Figure 1. The primary purpose of the research is to investigate the effects of the amount of OSep on the mechanical performance, crystallinity, morphology, thermal stability and shrinkage rate. The traditional PP/elastomer/talc composite is also made using the same process as a counterpart.

## 2. Materials and Methods

### 2.1. Materials

The polypropylene (PP) used in this work was purchased from China Petroleum and Chemical Commercial (MFR = 60 g/10 min at 230 °C/2.16 kg). Polyolefin elastomer (POE, MFR = 1 g/10 min at 190 °C/2.16 kg) was purchased from DOW. Unmodified sepiolite (Sep) and organically modified sepiolite (OSep) by cetyl trimethyl ammonium bromide (CTAB) were kindly supplied by Tolsa. The sepiolite has a diameter of about 20 nm and a length of 200–2000 nm. Talc (D50 = 2 μm) was purchased from Imerys.

### 2.2. Preparation of PP/OSep Nanocomposites

Organic modified sepiolite (OSep) was dried in a vacuum oven for 4 h at 120 °C. According to the formulation of PP/OSep nanocomposites (Table 1), PP/OSep nanocomposites with different OSep contents (0, 2.0 wt%, 4.0 wt%, 6.0 wt% and 8.0 wt%) were prepared by melt compounding using a twin-screw extruder (PLASTIC-ORDER, Brabender, Germany) with a screw speed of 500 rpm at 170–210 °C. The obtained PP/OSep nanocomposites were designated as PP/OSep-0, PP/OSep-2, PP/OSep-4, PP/OSep-6 and PP/OSep-8, respectively. PP/OSep nanocomposites were subsequently injection molded to characterize their properties.

### 2.3. Characterization

The chemical structures of unmodified sepiolite and organic modified sepiolite were analyzed with a Fourier transform infrared spectrophotometer (FTIR, Perkin Elmer, 2200, Waltham, MA, USA) with the wave number ranging from 4000 to 400 cm^−1^.

XRD patterns of unmodified sepiolite, organic modified sepiolite and PP/sepiolite nanocomposites were obtained using an X-ray diffractometer (XRD, Bruker, D8 advance, Karlsruhe, Germany). The scanning of the 2*θ* angle was from 5 to 30°. The scanning speed was 0.1 s/step in steps of 0.02°.

The thermal stability of PP/sepiolite nanocomposites was measured in a temperature range of 40–750 °C using a thermogravimetric analyzer (TGA, Netzsch, STA-449C-Jupiter, Bavaria, Germany) at a constant heating rate of 20 °C/min and under nitrogen atmosphere with a gas flow rate of 20 cm^3^/min.

The thermal properties of PP/sepiolite nanocomposites were measured using a differential scanning calorimeter analyzer (DSC, TA, Q20, New Castle, DE, USA). We heated 5–10 mg of the specimens hermetically sealed in an aluminum pan to 200 °C with a rate of 10 °C/min and held for 3 min to keep a consistent thermal history for the melting process. Then, the samples were cooled to 40 °C at a rate of 10 °C/min. The second heating was performed following the same heating rate up to 200 °C.

The flexural tests of PP/sepiolite nanocomposites were carried out using a universal experimental machine (SANS, UTM 6103, Shenzhen, China) according to ISO-178 at a crosshead speed of 2 mm/min and a span width of 64 mm at room temperature. The size of specimens is 80 mm × 10 mm × 4 mm. All data were the average of five independent measurements.

The Izod notched impact tests of PP/sepiolite nanocomposites were performed on a pendulum impact tester (SANS, ZBC2000, Shenzhen, China) according to ISO-180. The size of the specimens is 80 mm × 10 mm × 4 mm with a neck depth of 2 mm. All data were the average of five independent measurements.

The melting flow rate (MFR) of PP/sepiolite nanocomposites was tested using a melt flow rate instrument (SANS, MTM1000, Shenzhen, China) according to ISO-1133. The test temperature is 230 °C and the load is 2.16 kg.

The impact fracture morphology of PP/sepiolite nanocomposites was evaluated using a scanning electron microscope (SEM, HITACHI, S-3400N, Tokyo, Japan) with an accelerating voltage of 10 kV. SEM observations were performed after sputtering the samples with a thin film of gold for 40 s. Before sputtering the surface of samples, the samples were immersed in toluene for 48 h, and then baked at 80 °C for 30 min to remove residual toluene on the surface of the sample.

Capillary rheometry was used to understand the high shear viscosity of the PP/OSep nanocomposites using a capillary rheometer (RG20, Goettfert, Buchen, Germany). The diameter and length–diameter ratio of the capillary were 30 mm and 30:1, respectively. The shear rates were varied from 10 to 10,000 s^−1^ at 230 °C.

Rotational rheology measurements were carried out on a rotary rheometer (TA Instruments, ARES-G2, New Castle, USA) equipped with a parallel plate geometry using 25 mm diameter plates at 200 °C and in the angular frequency (ω) range from 0.01 to 100 rad/s under a strain amplitude of 5%.

The shrinkage rate of PP/sepiolite nanocomposites was evaluated using a rectangular plate mold with a length of L_O_ = 200 mm and a width of W_O_ = 50 mm. PP/sepiolite nanocomposites were injection molded to obtain the actual length L and width W of the rectangular plate. The shrinkage in the melt flow direction (FD) can be calculated using the equation S(FD) = (L − L_O_)∗100%/L_O_. The shrinkage perpendicular to the melt flow direction (TD) can be calculated using the equation S(TD) = (W − W_O_)∗100%/W_O_.

## 3. Results and Discussion

### 3.1. Chemical Structure

**Figure 2:**
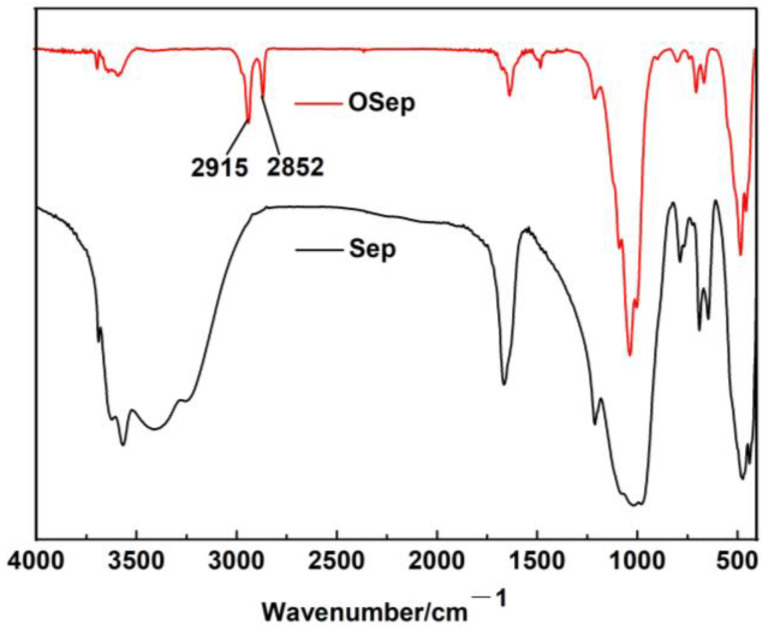
FT-IR spectra of Sep and OSep.

Figure 2 shows the FT-IR spectra of Sep and OSep. The coordinated water correspondence of the O-H stretching bond appears at 3569 cm^−1^ and O-H stretching deformation of zeolitic water bands for sepiolite is observed at 1664 cm^−1^ [22]. The Si-O coordination bonds at 1208 and 1019 cm^−1^ are observed as a result of the Si-O vibrations [23]. OSep has the new absorption peaks at 2915 and 2852 cm^−1^ compared to Sep, which are assigned to the asymmetric stretching vibrations and symmetric stretching vibrations of -CH_2_, respectively. This indicates that Sep is organically modified by cetyl trimethyl ammonium bromide (CTAB).

### 3.2. Morphology

**Figure 3:**
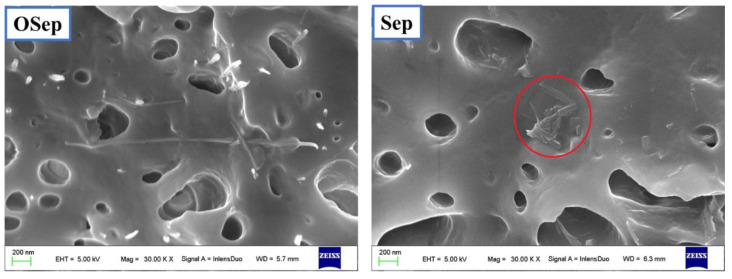
Morphology of PP/OSep-4 and PP/Sep-4 nanocomposites. It is the sepiolite in the red circle.

The dispersion of OSep particles and Sep particles in PP/sepiolite nanocomposites are observed by SEM. The morphology of PP/OSep-4 and PP/Sep-4 nanocomposites with 4 wt% sepiolite is shown in Figure 3. The OSep appears to disperse homogenously in the PP matrix without visible aggregates. However, there are big Sep aggregations in PP/Sep nanocomposite. Therefore, the organic modified sepiolite is a better choice for preparing the PP/sepiolite nanocomposites.

**Figure 4:**
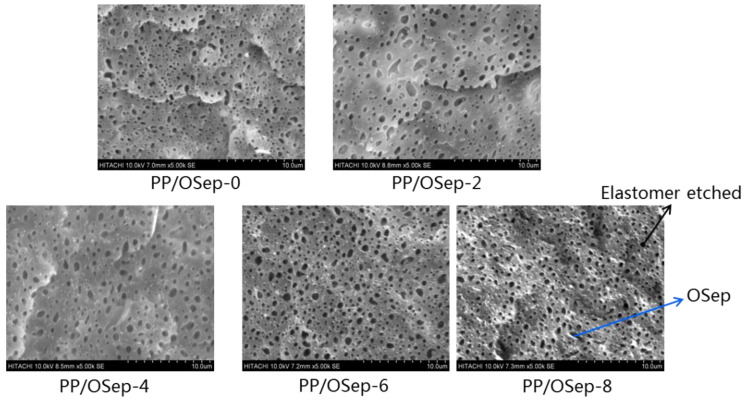
Morphology of PP/Osep nanocomposites with various Osep loadings.

Figure 4 shows the morphology of PP/OSep nanocomposites with various OSep loadings. With the increase of OSep loading, the number of OSep particles increases and the OSep particles still disperse evenly without any aggregates. This indicates that the OSep particles disperse easily because of their good compatibility with PP. This is because that the organic modified sepiolite has alkyl macromolecular chains, which can increase the physical adsorption and the bonding force on the interfaces between sepiolite and PP. The voids are caused by the remove of elastomers by toluene etching. So, the morphology and size of the void are equal to the morphology and size of the elastomer. The size of void first increases and then decreases with the increase of Osep, indicating that the elastomers first become bigger and then become smaller.

### 3.3. Rheology

**Figure 5:**
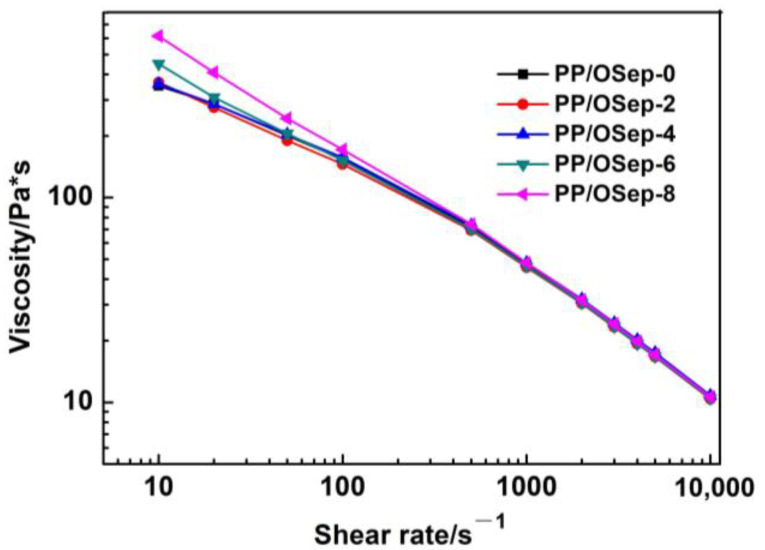
Viscosity of PP/OSep nanocomposites with different OSep contents.

The rheological analysis allows us to check the processability of proposed formulations to see if they are suitable for industrial processing. The rheological properties of PP/OSep nanocomposites with different OSep contents were investigated by capillary rheometer, as shown in Figure 5. As expected, the viscosity of PP/OSep nanocomposites gradually reduces as the shear rate increases. Under low shear rate (<100 s^−1^), the viscosity of PP/OSep nanocomposites first decreases and then increases with the increase of OSep content, and PP/OSep-2 shows a lowest viscosity. This may be attributed to the plasticizing effect of the organic surfactant on the filler surface [24]. The increases in viscosity of PP/OSep nanocomposites may be because of the enhanced interaction between the OSep particles and PP molecular chain with the increase of OSep content. The viscosity difference of PP/OSep nanocomposites will change the size of elastomers. The higher the PP viscosity, the smaller the elastomer. The viscosity of PP/OSep nanocomposites first decreases and then increases, which will make the size of elastomer first increase and then decrease. The results are in line with the morphology observed in Figure 4.

The fluidity of plastics is usually described by the melting flow rate (MFR) at a certain temperature and load. With the increase of OSep content, the MFR of PP/OSep nanocomposites first increases and then decreases (Table 2). This increase of MFR may be due to the low molecular weight organic surface modifier of OSep which plays the lubricating agent role. On the other hand, the inhibited crystallinity of PP by OSep improves the fluidity of PP materials. When the content of OSep is high, the MFR of PP/OSep nanocomposite decreases. This may be attributed to the interaction between the PP molecular chain and OSep and the network structure of OSep clays, which increase the viscosity and hence decrease the fluidity.

The shear rate (γ_w_) of the melt at the capillary wall can be calculated according to formula γ_w_ = −dυ/dr = 4 Q/(πR^3^). In the equation, R is the capillary radius (m), L is the capillary length (m) and Q is the volume flow rate (m^3^/s). This formula can be used to calculate the shear rate of PP/OSep nanocomposites with different OSep contents when measuring the melt flow rate. The diameter of the die used is 2.095 ± 0.005 mm. The melt density of PP is 0.7386 g/cm^3^. Based on the MFR data in Table 2, the shear rates of PP/OSep nanocomposites with various OSep contents are calculated: 64.6 s^−1^ (PP/OSep-0), 76.9 s^−1^ (PP/OSep-2), 70.1 s^−1^ (PP/OSep-4), 55.1 s^−1^ (PP/OSep-6) and 44.6 s^−1^ (PP/OSep-8). For the capillary viscosity based on the shear rate between 40 and 80 s^−1^, the viscosity of PP/OSep nanocomposites with 2 wt% OSep reduces, which improves the fluidity and MFR. The viscosity of PP/OSep nanocomposites with 4 wt% OSep is lower than that of unfilled PP material. With the addition of 8 wt% OSep, the viscosity, fluidity and MFR of PP composite increase significantly. The data of capillary rheology agrees well with the change of MFR.

**Figure 6:**
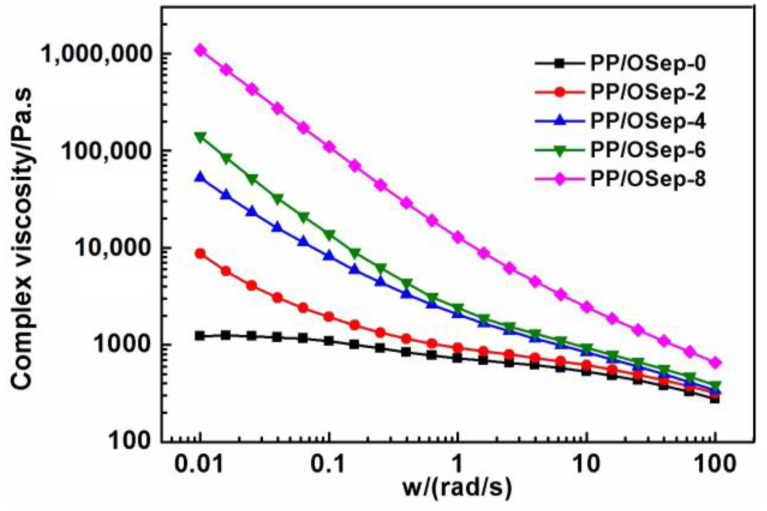
Complex viscosity of PP/Osep nanocomposites with different Osep contents.

**Figure 7:**
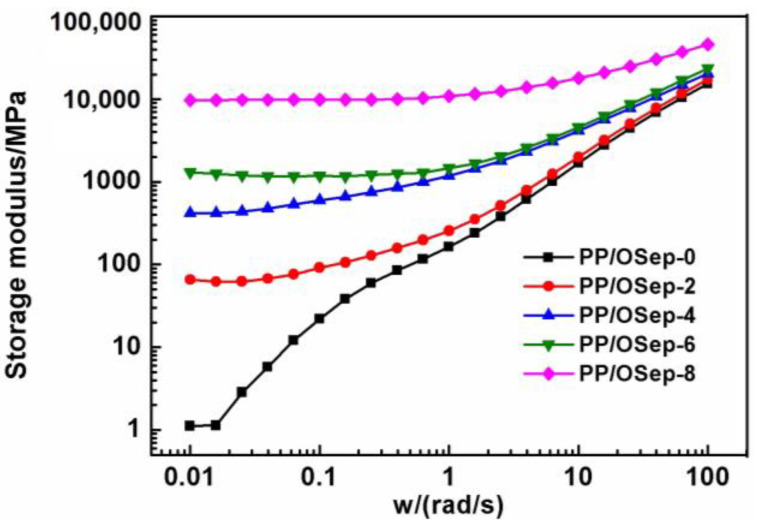
Storage modulus of PP/Osep nanocomposites with different Osep contents.

**Figure 8:**
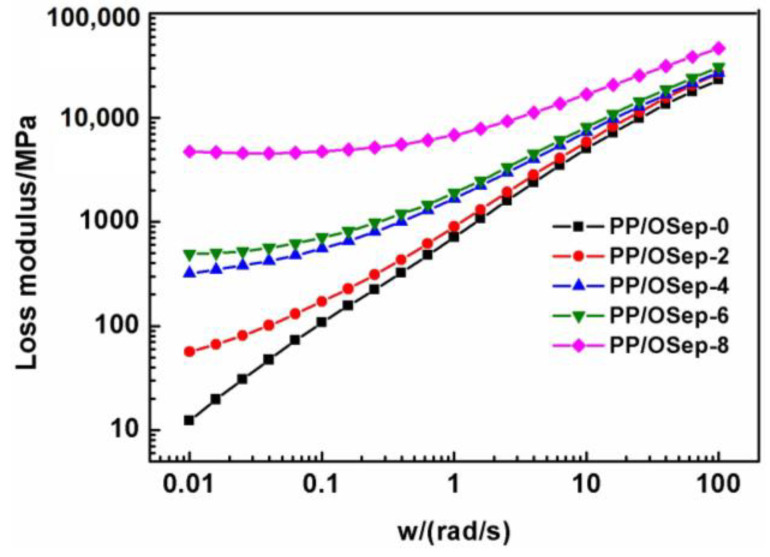
Loss modulus of PP/Osep nanocomposites with different Osep contents.

The rotational rheological properties of PP/OSep nanocomposites with various OSep loadings are presented in Figure 6, Figure 7 and Figure 8. The complex viscosity of PP/OSep nanocomposites increases with the increase of OSep (Figure 6). In general, the addition of rigid fillers in a soft matrix acts as a reinforcement and restricts the mobility of surrounding polymer chains, leading to the increase of melt viscosity. In particular, the increase extent of melt viscosity is enhanced in the presence of nanoparticles with an increased surface area to volume ratio if the nanoparticles are well dispersed in the matrix compared with the micron sized fillers [15]. The increase in complex viscosity is a good indication of a fine dispersion of Osep in PP matrix.

The storage modulus (G′) and loss modulus (G″) of PP/Osep nanocomposites are shown in Figure 7 and Figure 8, respectively. Both the storage modulus and loss modulus show a monotonic increase with increasing Osep loading at the scanning range, especially at the low frequency region. However, the storage modulus and loss modulus of PP/OSep nanocomposites are equivalent at high frequency. The increase of the storage modulus may be due to the confinement of polymer chains caused by the strong interactions between the uniformly dispersed OSep particles and PP matrix [18,25,26,27,28,29]. The increase of storage modulus is attributed to stiffness imparted by the solid OSep fibers. The confinement of polymer chains by the OSep fiber decreases with the increase of angular frequency. Therefore, it is considered that the fiber–fiber interactions of OSep give a great devotion to storage modulus at low frequency region, but the polymer matrix contribution becomes significant to storage modulus at high frequency region.

### 3.4. Crystallinity

**Figure 9:**
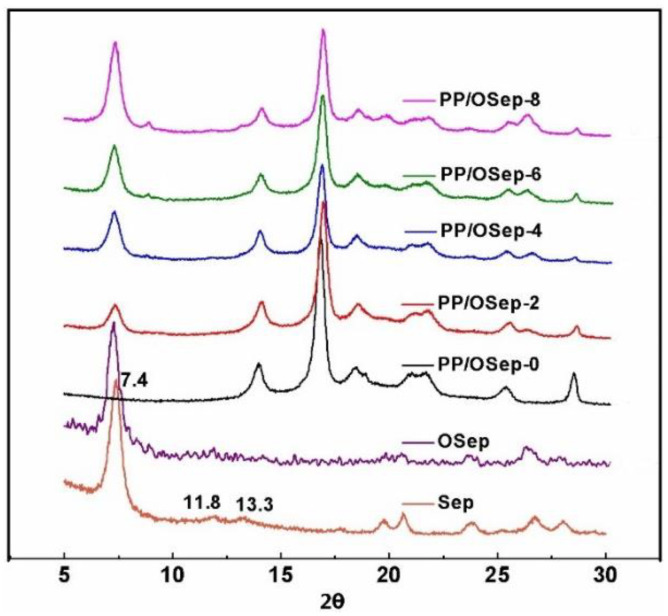
XRD patterns of Sep, Osep and PP/OSep nanocomposites.

The crystallinity of Sep, OSep and PP/OSep nanocomposites containing different OSep contents was investigated by XRD, as shown in Figure 9. The peaks at 2*θ* = 7.4, 11.8 and 13.3° correspond to the primary diffraction of the (110), (200) and (400) planes of the Sep, respectively. The characteristic peak of OSep is also at 2*θ* = 7.4°. The XRD pattern of PP shows five major peaks in the 2*θ* range of 5–30° without any peculiar peak corresponding to beta (β) and gamma (γ) form of PP crystals, confirming the presence of only monoclinic α-form of PP crystals. In PP/OSep nanocomposites, the peak intensity corresponding to 2*θ* = 7.2° increases with increasing OSep concentration. It is also observed that the intensity of the peak at 2*θ* = 14.0° and 2*θ* = 16.9°, corresponding to (040) plane and (110) plane of a-phase, respectively, decreases with increasing OSep loading, showing a decrease of crystallinity.

**Figure 10:**
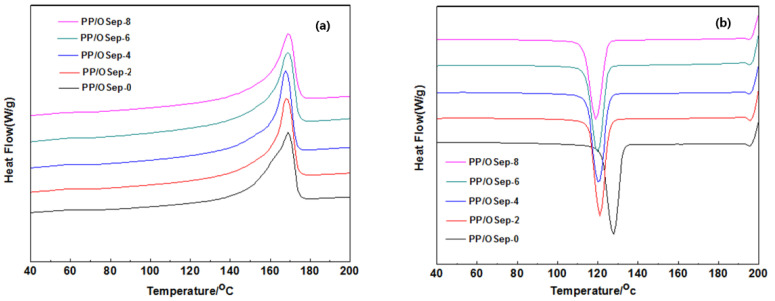
Second heating curves (**a**) and cooling curves (**b**) of PP/OSep nanocomposites with different OSep contents.

**Figure 11:**
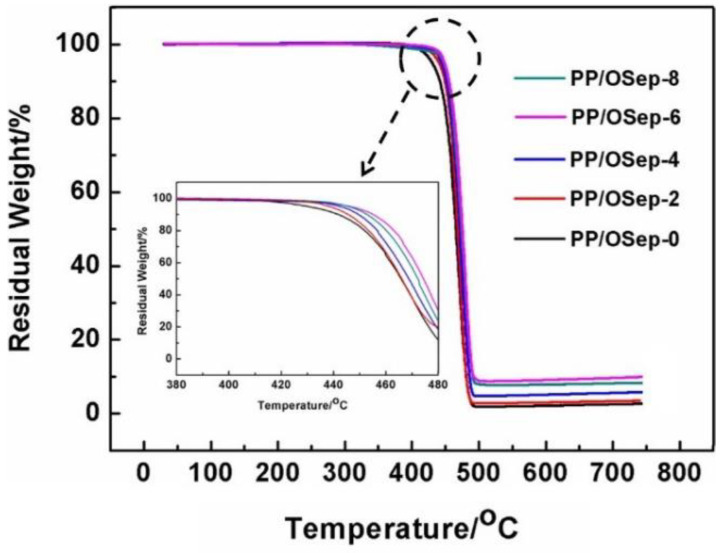
TG curves of PP/OSep nanocomposites with different OSep contents.

The crystallinity of PP/OSep nanocomposites is also investigated by differential scanning calorimetry (DSC). The second heating curves and cooling curves are shown in Figure 10 and Figure 11, respectively. Table 3 shows the DSC scan results of PP/OSep nanocomposites. It can be seen that both the melting enthalpy (ΔHm) and crystallization temperature (Tc) decrease with the increase the OSep content, implying that the OSep fiber restrains the crystallization of polypropylene. These results are consistent with the XRD ones.

### 3.5. Thermal Stability

The thermal stability of PP/OSep nanocomposites was evaluated using thermogravimetric analysis (TGA) in a nitrogen atmosphere. The temperature (T_5%_) corresponding to 5 wt% weight loss is defined as the initial decomposition temperature. The TGA curves are shown in Figure 11. The T_5%_ of PP/OSep nanocomposites increases with the OSep content, indicating the improvement in the thermal stability. Meanwhile, more OSep is used, more residue content of PP/OSep nanocomposites is found at 650 °C. These results indicate that the thermal stability of PP/OSep nanocomposites increases with the amount of OSep. This can be attributed to the strong interaction between OSep and PP, which can restrict the movement of polymer molecular chains, and the OSep can hinder the heat transfer and the volatilization of the decomposition products.

### 3.6. Mechanical Properties

It is known that a homogeneous dispersion of nano-fillers in a polymer matrix provides maximum reinforcement via load transfer and deflection of cracks. The mechanical properties of nanocomposites are directly related to the homogeneous nano-filler dispersion and intercalation in the polymer matrix. Interactions between the nano-filler and the polymer matrix lead to a high flexural modulus. The mechanical properties of PP/OSep nanocomposites with different OSep contents are listed in Table 4.

As the OSep content increases, the Izod notched impact strength of PP/OSep nanocomposites first decreases and then increases. As previously described in the morphology observation of PP/OSep nanocomposites, the size of elastomers first increases and then decreases with the increase of OSep content. The impact strength depends on the size of elastomers. The smaller the elastomers, the higher the impact strength. Therefore, the change in the impact strength is attributed to the initial increase and subsequent decrease of elastomer size.

The flexural modulus of PP/OSep nanocomposites significantly increases with increasing OSep content. This significant improvement in flexural modulus can be attributed to the good dispersion of the OSep particles with high aspect ratio and high intrinsic stiffness, as well as the strong interaction between OSep fiber and PP matrix [30].

### 3.7. Mold Shrinkage

**Figure 12:**
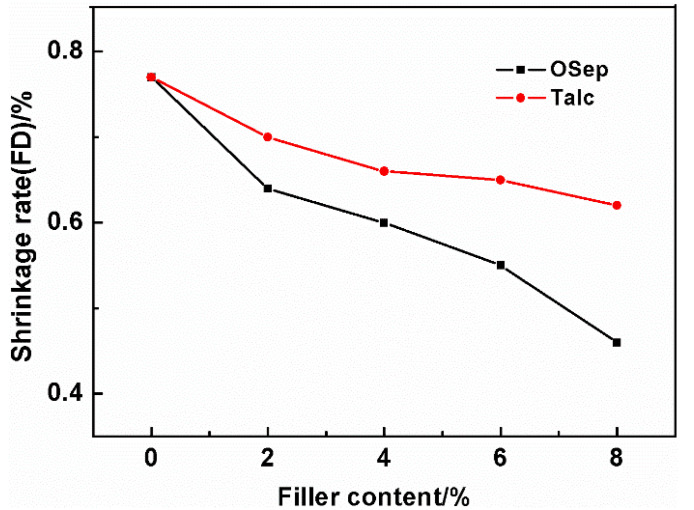
Shrinkage rate (FD) of PP materials with different filler contents.

**Figure 13:**
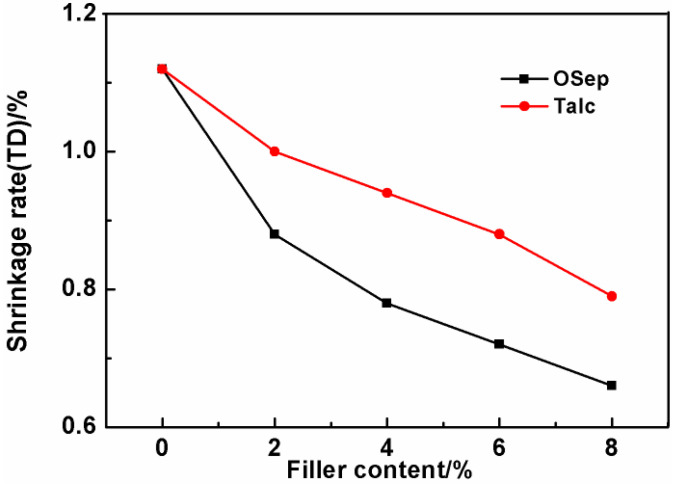
Shrinkage rate (TD) of PP materials with different filler contents.

PP is a semi-crystalline polymer with a high mold shrinkage, which limits its usage in some applications requiring low shrinkage of plastic parts. In order to reduce the shrinkage rate of PP materials, glass fibers (GF) and mineral fillers are commonly used. However, mostly the shrinkage rate decreases more in parallel to the flow direction (FD) than perpendicular to the flow direction (TD), leading to warpage and distortion. This limits its further applications, especially with respect to large automotive parts. Therefore, the effects of OSep and talc powder with different contents on the FD and TD shrinkage rate of PP materials are investigated. The results are shown in Figure 12 and Figure 13. With the increase of OSep content, the FD and TD shrinkage rates gradually decrease and become closer. Because OSep itself does not shrink, and the addition of OSep reduces the crystallinity of PP material, the crystallization shrinkage of PP materials is reduced. The OSep has a diameter of about 20 nm and a length of only 600 nm. Hence, the shrinkage rate in different directions does not differ much. Compared with talc under the same loading, OSep reduces the shrinkage by about twice as much as talc. In other words, the shrinkage rate of PP/OSep nanocomposites can reach the equivalent shrinkage rate of talc–filled PP materials containing twice the talc filler content, which is very important for the dimensional accuracy of low-density materials. The low-density PP/OSep nanocomposites can replace the talc filled PP materials used in the existing molds.

## 4. Conclusions

In this study, PP/sepiolite nanocomposites were prepared via the melt blending method. The dispersion of nano-sepiolite and its effect on the properties of PP materials were investigated. The results showed that the organic modified nano-sepiolite (OSep) had good compatibility with PP and could be evenly dispersed in PP matrix. The addition of OSep improved the storage modulus and thermal stability, showing a strong interaction between OSep and PP matrix. With the increase of Osep content, the fluidity of PP/Osep composites first increased due to the lubrication of surface modifiers and then decreased due to the interaction between OSep and PP. The viscosity ratio between the toughening agent elastomer and PP first increased and then decreased. Therefore, the size of the dispersive phase elastomer first increased and then decreased, and the impact notched strength of PP/Osep composites first decreased and then increased. Due to the interaction between OSep and the PP molecular chain, the crystallization of PP was restricted. Hence, the loading of OSep reduced the crystallinity and the shrinkage rate of PP. OSep is suitable for the preparation of high-performance lightweight PP composites. PP/OSep nanocomposites can potentially replace the talc filled PP materials used in the existing molds to reduce the weight of automotive PP materials.

## Figures and Tables

**Table 1:** Formulation of PP/OSep nanocomposites.

Samples	PP/OSep-0	PP/OSep-2	PP/OSep-4	PP/OSep-6	PP/OSep-8
PP	80	78	76	74	72
POE	20	20	20	20	20
OSep		2	4	6	8

**Table 2:** Melting flow rate (MFR) of PP/OSep nanocomposites.

Samples	PP/OSep-0	PP/OSep-2	PP/OSep-4	PP/OSep-6	PP/OSep-8
MFR (g/10 min), 230 °C/2.16 Kg	25.8	30.7	28	22	17.8

**Table 3:** DSC scan results of PP/OSep nanocomposites.

Samples	PP/OSep-0	PP/OSep-2	PP/OSep-4	PP/OSep-6	PP/OSep-8
ΔHm (J/)	63.8	62.4	61.3	60.4	59.2
Tc (°C)	127.8	121	120.4	119.9	119.1

**Table 4:** Mechanical properties of PP/OSep nanocomposites.

Samples	Flexural Modulus (MPa)	Izod Notched Impact Strength (KJ/m^2^)
PP/OSep-0	1150	24.4
PP/OSep-2	1202	7.4
PP/OSep-4	1331	6.6
PP/OSep-6	1453	9.2
PP/OSep-8	1584	18.3

## Data Availability

No new data were created or analyzed in this study. Data sharing is not applicable to this article.
